# *Kadukkai maathirai* (Indian herbal drug) prevents hepatocellular cancer progression by enhancing GSTM1 expression and modulating β catenin transcription: in-silico and in-vivo study

**DOI:** 10.12688/f1000research.145961.2

**Published:** 2024-10-22

**Authors:** Manjunath Shetty, Smita Shenoy, Arul Amuthan, Vasudha Devi, Nitesh Kumar, Amruth Kiran, Ganesh Shenoy, Diya Rajasekhar Chinta, Shama Prasada K, Akshatha Shetty, Mohandas Rao K G

**Affiliations:** 1 Centre Of Excellence, Ocular Nanoscience, Manipal Academy of Higher Education, Manipal, Karnataka, 576104, India; 2 Division of Pharmacology, Department of Basic Medical Sciences, Manipal Academy of Higher Education, Manipal, Karnataka, 576104, India; 3 Department of Pharmacology, Kasturba Medical College, Manipal, Manipal Academy of Higher Education, Manipal, Karnataka, 576104, India; 4 Department of Pharmacology and Toxicology, National Institute of Pharmaceutical Education and Research, Vaishali, Bihar, 844102, India; 5 Department of Pharmacology, Manipal University College Malaysia, Bukit Baru, Melaka, 75150, Malaysia; 6 Department of Cell and Molecular Biology, School of Life Sciences, Manipal Academy of Higher Education, Manipal, Manipal, Karnataka, 576104, India; 7 Department of Research and Development, Muniyal Institute of Ayurveda and Medical Sciences, Manipal, Manipal, Karnataka, 576104, India; 8 Division of Anatomy, Department of Basic Medical Sciences, Manipal Academy of Higher Education, Manipal, Karnataka, 576104, India

**Keywords:** β catenin, Glutathione-S transferase mu 1, Siddha, herbal, Diethyl nitrosamine

## Abstract

**Background:**

Hepatocellular carcinoma (HCC) is an aggressive malignancy with poor clinical outcomes. Hence cost-effective drugs with fewer side effects as a standard supportive therapy might yield substantial advantages in efficacy and safety. *Kadukkai maathirai* (KM) is being used as a supplement in hepatocellular carcinoma. We evaluated whether KM has any preventive action on cancer progression in diethyl nitrosamine (DEN) - induced HCC in rats.

**Methods:**

DEN was injected to produce HCC in rats, which was confirmed after 16 weeks. All the rats were orally administered KM for 4 weeks. Hepatoprotective potential (serum AST, ALT, ALP, Bilirubin) and anticancer efficacy (body weight, nodule count, tumor progression by histopathology, expression of GSTM1 by Liquid chromatography-mass spectrometry (LC-MS), and In-silico analysis of phytoconstituents against β catenin and LRP analysis were evaluated.

**Results:**

KM prevented cancer progression against DEN-induced HCC by an increase in GSTM1, a phase II detoxifying enzyme. It significantly reversed altered nodule count, relative liver weight, body weight, and histopathological features of HCC. *In silico* analysis of phytoconstituents of KM showed that they modulate the intracellular transcription process by inhibiting the armadillo repeat region of β catenin.

**Conclusions:**

Our results elucidate the potential of KM as a supplement in HCC by reducing nodule count, protecting the liver from further damage, GSTM1 expression, and inhibiting armadillo repeat region of β catenin.

## Introduction

Hepatocellular carcinoma (HCC) is among the sixth most common and the second most lethal cancers. ^1^ It may occur as a sequel to chronic liver conditions such as alcohol and tobacco consumption, obesity, and infections like hepatitis B, hepatitis C, and aflatoxins. ^2^^,^^3^ These factors lead to liver inflammation and fibrosis, followed by the destruction of the normal liver architecture.

Chemotherapy, radiotherapy, radiofrequency ablation, hepatectomy, and transplantation are the management plans of HCC. ^4^ However, the survival rate is low, suggesting the need for improved therapy. ^5^ Chemotherapeutic agents used include sorafenib (MAP-kinase inhibitor), doxorubicin, 5-fluorouracil (5-FU), and cisplatin. ^6^ Being a multikinase inhibitor sorafenib, acts on vascular endothelial growth factor (VEGF) receptors, and prolongs survival so this drug is considered as a new reference standard for advanced HCC. However, it elicits adverse effects including skin toxicities, diarrhea, and hypertension, along with other general toxicities like anorexia, hair loss, weight loss, etc. ^7^

Compared to conventional modes of treatment, plant-based medicines have generally fewer side effects, are cost-effective, and well tolerated by patients. ^8^ *Kadukkai maathirai* (KM) is an Indian traditional medicine prescribed by Siddha practitioners to treat edema in severe anemia and ascites secondary to liver disease. KM consists of *Terminalia chebula Retz.* (Combretaceae *), Piper nigrum L.* (Piperaceae) *, Eclipta alba L.* (Asteraceae), *Citrus L.* (Rutaceae) and ferrous sulfate. ^9^ Each of the constituents mentioned is known to have a hepatoprotective effect. ^10^ *T. chebula* consists of gallic acid and chebulic acid; gallic acid has anti-inflammatory and antioxidant properties, whereas chebulic acid has antioxidant with hepatoprotective properties. ^11^ *P. nigrum* inhibits lipid peroxidation and superoxide free radical generation due to its antioxidant effect exerted by phenols and flavonoids. ^12^^,^^13^ Coumestans, present in *E. alba*, stimulates the regeneration of hepatocytes and protect the liver. ^14^

Though *Kadukkai maathirai* is used for treating liver disorders by practitioners of *Siddha* medicine, yet its effect on hepatocellular carcinoma has not been explored. Diethylnitrosamine (DEN) is a known potential hepatocarcinogen that disrupts nuclear enzymes that help in the repair of DNA and replication. ^14^ In our previous study we demonstrated that the KM at the dose of 144 mg/kg treatment exhibited significant protection which was evident both by histology and biochemically against D-galactosamine-induced hepatic necrosis in rats. ^15^

Hepatic DEN metabolism produces O6-ethyl deoxyguanosine and O4- and O6-ethyl deoxythymidine, which are mutagenic and carcinogenic. ^16^^,^^17^ The effect of analyzed phytoconstituents of KM on low-density lipoprotein (LDL) receptor-related protein-6 (LRP6) and β-catenin signaling pathway, which play a role in hepatocellular carcinoma (HCC), was also studied *in silico.*

## Methods

### Chemicals and consumables

Diethylnitrosamine 0.01% (Catalogue number - D0516) from TCI chemicals, Tokyo, Japan, assay kits for liver function test from Spain-based Spinreact, silymarin 50mg/kg body weight/day (Item Number – 46791) from Micro Labs Limited, India, *Kadukkai maathirai* (MMP14013) *from* India-based SKM *Siddha* and Ayurveda (GMP certified) Company (India) Ltd. Good laboratory-grade chemicals were used.

### Animals

**Ethical statement:** Seven-week-old female Sprague Dawley rats (150-200 g), procured from the Central Animal Research Facilities (CARF) of Kasturba Medical College, Manipal Academy of Higher Education, Manipal were used in the study after getting approval from the Institutional Animal Ethics Committee approval (IAEC/KMC/19/2016 dated 16.03.2016). Guidelines given by the Committee for Control and Supervision of Experiments on Animals (CCSEA), Government of India, New Delhi for the use of laboratory animals were followed for the maintenance of animals. ^18^ All efforts were made to ameliorate suffering of animals. All the rats were observed daily to check their health and ensure continuous access to food and water. Bedding in the cages were changed daily to provide healthy living conditions. The location of the animal cage was not changed throughout the study. The rats were acclimatized and maintained at 27 ± 3°C, humidity of 60 ± 10%, and a 12 h light/dark cycle. No procedures were done which would cause sustained pain. Anesthetics and the method of euthanasia used was as per guidelines of CCSEA.

**Reporting Guidelines:** The authors confirm that they followed and adhered to ARRIVE 2.0 Checklist for their study. ARRIVE CHECKLIST [Internet]. figshare; 2024 [cited 2024 Jun 7]. Available from: https://figshare.com/articles/dataset/ARRIVE_CHECKLIST/25910563/1. ^76^

**Study design:** The rats were randomised into 2 groups based on their body weight: normal control (n=6) and toxic control (n=30) rats.

**Induction of liver cancer:** The number (n=6) of animals in each group was based on earlier study. ^19^ The groups were as follows: The normal control (group 1) consisted of a total of 6 rats and received drinking water for 16 weeks. The toxic control group (DEN) consisted of a total of 30 rats, which received DEN 0.01% for sixteen weeks through drinking water for induction of HCC. ^20^ At the end of 16 weeks, animals were divided into 5 groups (n=6/group) and received treatment orally as follows. Group 2- toxicant control and received gum acacia 2% 1 mL/kg orally for 4 weeks. ^21^ Group 3, 4, 5- test drug KM36, 72, 144 mg/kg body weight orally for 4 weeks respectively. ^22^ Group 6- received Silymarin (50 mg/kg body weight) orally for 4 weeks. ^23^

At the end of the study (total of 20 weeks) blood was drawn by retroorbital puncture. The animals of all groups were euthanised by administering pentobarbitone intraperitoneally in a dose of 200 mg/kg body weight, ^24^ then livers were dissected for histopathology and polyacrylamide gel electrophoresis.

### Parameters assessed

• **Parameters to evaluate hepatoprotective potential of KM**
  After 4 weeks of KM treatment, under ketamine anesthesia (50 mg/kg i.p), ^25^ blood was withdrawn from all the rats from each group to estimate the serum AST, ALT, ALP, bilirubin, and total protein levels using kits (Aspen Laboratory). ^26^
• **Parameters to evaluate whether KM has any anticancer effect**

**Determination of liver weight and nodule count**

The liver of each animal was weighed and relative weight was calculated. The nodules on the liver were counted, and an intergroup comparison was made.

**Determination of body weight**

Weight was measured at the end of study by using a metis electronic weighing balance. The weight difference was calculated.

**Histopathological evaluation**

10% formalin was used as the fixative. Paraffin-embedded wax blocks were prepared and the tissue section was prepared at 4μm thickness. The hepatic tissue was stained with hematoxylin-eosin and studied. ^27^ Regarding the animal group, the assessor was blinded.

**Polyacrylamide gel electrophoresis for the separation of proteins and mass spectrometry for the identification of proteins.**

Sample Preparation and Electrospray-ionisation Quadrapole time of fight mass (ESI/QTOF) liquid chromatography and mass spectrometry analysis (LC-MS). Tissue extract was prepared, and proteins were separated by polyacrylamide gel electrophoresis. LC-MS analysis was used to identify the protein that was differentially expressed in response to DEN administration. The expressed protein was identified by coomassie staining, separated from PAGE gel, and subjected to LC-MS analysis using MASCOT Ver 2.3. ^28^

**In-silico analysis of phytoconstituents of KM against β catenin and LRP6 in HCC**

The binding mode and interaction of β catenin and LRP6-E3 with each of the derived list of phytoconstituents of *Kadukkai maathirai* was performed using AutoDock Vina software. ^29^^,^^30^ Grid boxes were set at two binding sites of the armadillo repeat region of β catenin and their XYZ coordinates include X: 107.640 Y: -29.892 Z: 1.991 Å and X: 102.640 Y: 9.850 Z: 29.741 Å respectively with a grid size of 40. Whereas the XYZ coordinates for LRP6-E3 were defined by establishing a grid box with the dimensions of X: -29.376 Y: 20.133 Z: -10.560 Å with a grid size of 40, The interactions of β catenin and LRP6 protein-ligand conformations, including hydrogen, and hydrophobic bonds along with their interacting residues were analyzed using BIOVIA Discovery Studio Visualizer. ^31^ The 2D structures of phytoconstituents present in KM were collected from the PubChem Compound database. A total of 19 structures were sketched, prepared, and saved in MDL mol format using ChemSketch 2019 2.2 ^32^ and saved as PDBQT format. The 3D structure of β catenin (PDB ID: 1JPW) and LRP6 (PDB ID: 3S8V) were retrieved from Protein Data Bank (RCSB PDB), optimized, minimized their energy and saved as PDB format.

**Analysis of extract using Gas chromatography-mass spectroscopy (GC-MS)**

It was done with Shimadzu -QP2010. Column (30m length) used was RTX-5. Interface temperature 280 °C and ion source was 200 °C. Sample was injected (1 μL) for analysis and flow rate retained at 1 mL/minute. MS spectrum achieved was identified and compared with NIST library for confirmation of compounds. ^33^

### Statistical analysis

The data of control, test drug control, and experimental groups were presented in the form of mean ± SD, and one-way ANOVA and post hoc Tukey's test (SPSS version 16) were performed. Statistical significance was considered at p<0.05.

## Results

### Hepatoprotective potential of KM

**Biochemical estimation in serum**

Serum AST, ALP, ALT, and TBIL in DEN alone treated animals increased significantly in comparison to normal control indicating liver damage ( Table 1). KM 36 mg/kg and KM72 mg/kg in DEN-treated groups showed a fall in the AST, ALT, ALP, and TBIL levels compared to animals that received DEN alone but it was not statistically significant. Other parameters were not affected significantly ( Table 1).

**Table 1.** Effect of KM on liver function in DEN-induced HCC model in rats.

Groups	Serum AST (U/L)	Serum ALT (U/L)	Serum ALP (U/L)	Serum TBIL (mg/dL)	Serum Albumin (g/dL)
**Normal control**	59.0±7.5	35.4±8.7	120.8±20.0	0.03±0.01	1.44±0.3
**DEN**	145.5±24.1 *	99.0±14.8 *	274.3±69.1 *	0.126±0.08 *	0.9±0.1
**DEN+KM36 mg/kg**	107.7±10.4	73.9±8.7	243.6±14.7	0.036±0.00	1.3±0.1
**DEN+KM72 mg/kg**	131.0±26.4	93.8±32.6	283.6±42.5	0.068±0.06	1.3±0.4
**DEN+KM144 mg/kg**	85.5±13.2 ^#^^,^^€^^,^^¥^	71.1±10.0	257.25±26.2	0.035±0.02	0.9±0.1
**DEN+Silymarin**	136.2±37.1	83.15±18.8	280.75±82.9	0.099±0.02	0.9±0.1

* p<0.05 versus control.

^#^ p<0.05 versus DEN.

^€^ p<0.05 versus DEN+KM72 mg/kg.

^¥^ p<0.05 versus DEN+Silymarin, (Values - Mean ±SD).

**Bodyweight/Organ weight/Nodule count:**

**Figure 1.**
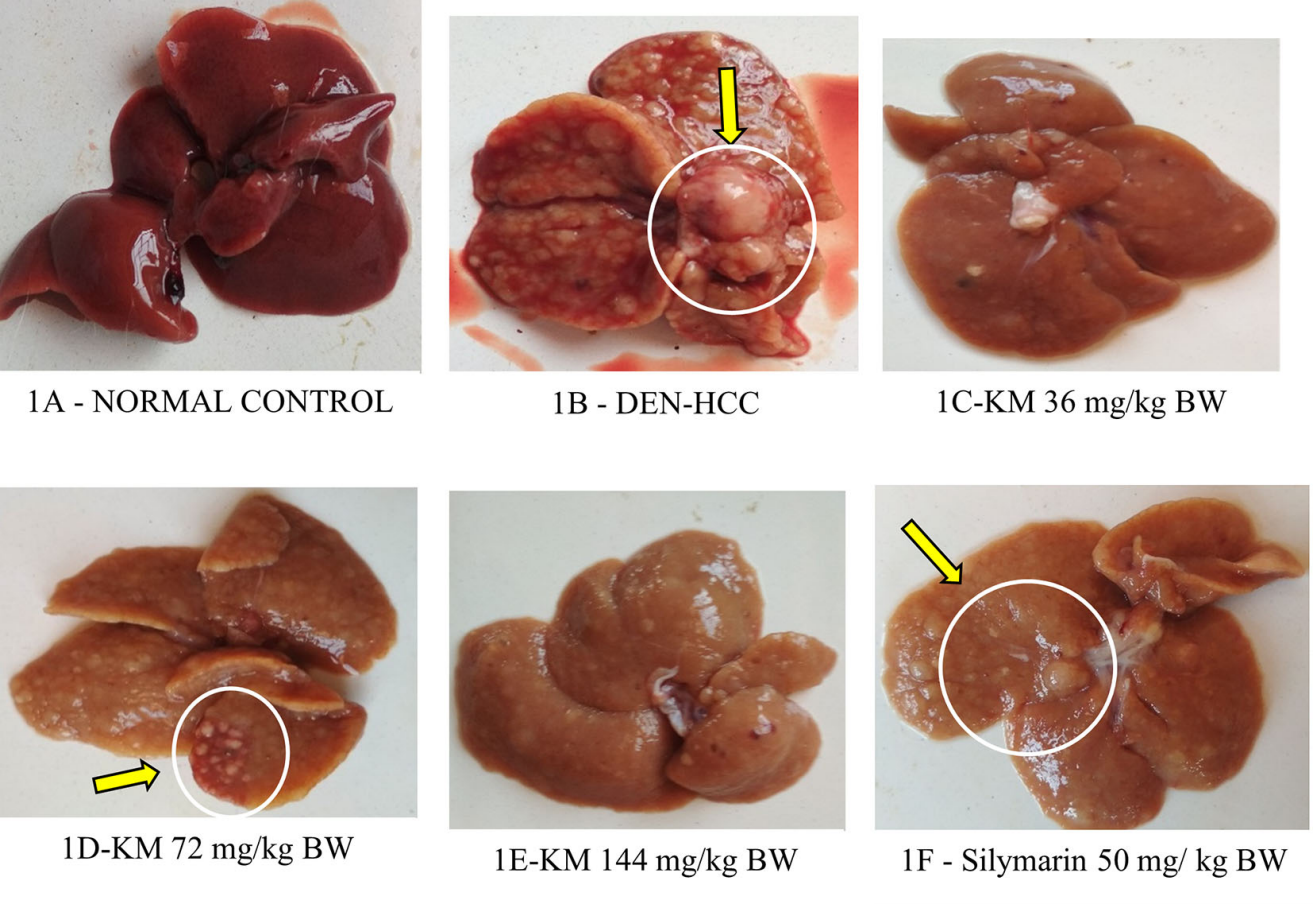
Effect of KM on the gross (macroscopic) appearance of liver in DEN-induced HCC model in rats. Yellow arrows indicate nodules. 1A (Normal control): Normal morphology of liver with smooth-surfaced hepatic lobes in the control animal. 1B (DEN): DEN alone administered group showed highly enlarged nodules with several foci on the surface of the liver and many greyish-white nodules, indicative of HCC. 1C (DEN +KM 36 mg/kg), 1D (DEN +KM 72 mg/ kg), 1E (DEN+KM 144 mg/kg), 1F (DEN + silymarin 50 mg/kg) groups showed relatively less foci on the hepatic surface and very few nodules when compared to 1B (DEN).

Body weight decreased significantly and relative liver weight increased in the DEN-induced HCC group versus control. These were altered favorably(p<0.05) in DEN-treated rats who received KM 36, 72, 144 mg/kg, and silymarin as compared to DEN-alone treated rats ( Table 2). A decrease (p<0.05) in nodule count in rats treated with KM 36 mg/kg, KM 72 mg/kg, and silymarin was observed in comparison to DEN group ( Table 2, Figure 1).

**Table 2.** Effect of KM on body weight, relative weight of liver and nodule count in DEN-induced hepatocellular carcinoma model in rats.

Groups	Nodule count	Relative weight of liver (g)	Bodyweight(g)
**Normal control**	0	0.02±0.002	257.5±8.8
**DEN (Diethylnitrosamine)**	107±4.0 *	0.09±0.017 ^*^	165.3±4.1 *
**DEN+KM 36 mg/kg**	72.0±8.1 ^#^^,^^€^	0.03±0.005 ^#^	222.6±9.5 ^#^
**DEN+KM 72 mg/kg**	84.4±11.8 ^#^	0.04±0.004 ^#^	200.2±3.7 ^#^
**DEN+KM 144 mg/kg**	97.5±2.6	0.04±0.005 ^#^	212.7±16.8 ^#^
**DEN+Silymarin**	59.7±4.1 ^#^^,^^¥^^,^^€^	0.04±0.007 ^#^	200.0±8.4 ^#^

* p<0.05 versus control.

^#^ p < 0.05 versus DEN.

^€^ p< 0.05 versus DEN+KM144.

^¥^ p<0.05 versus DEN+KM72. Values: Mean±SD.

### Histopathological evaluation of the liver

**Figure 2.**
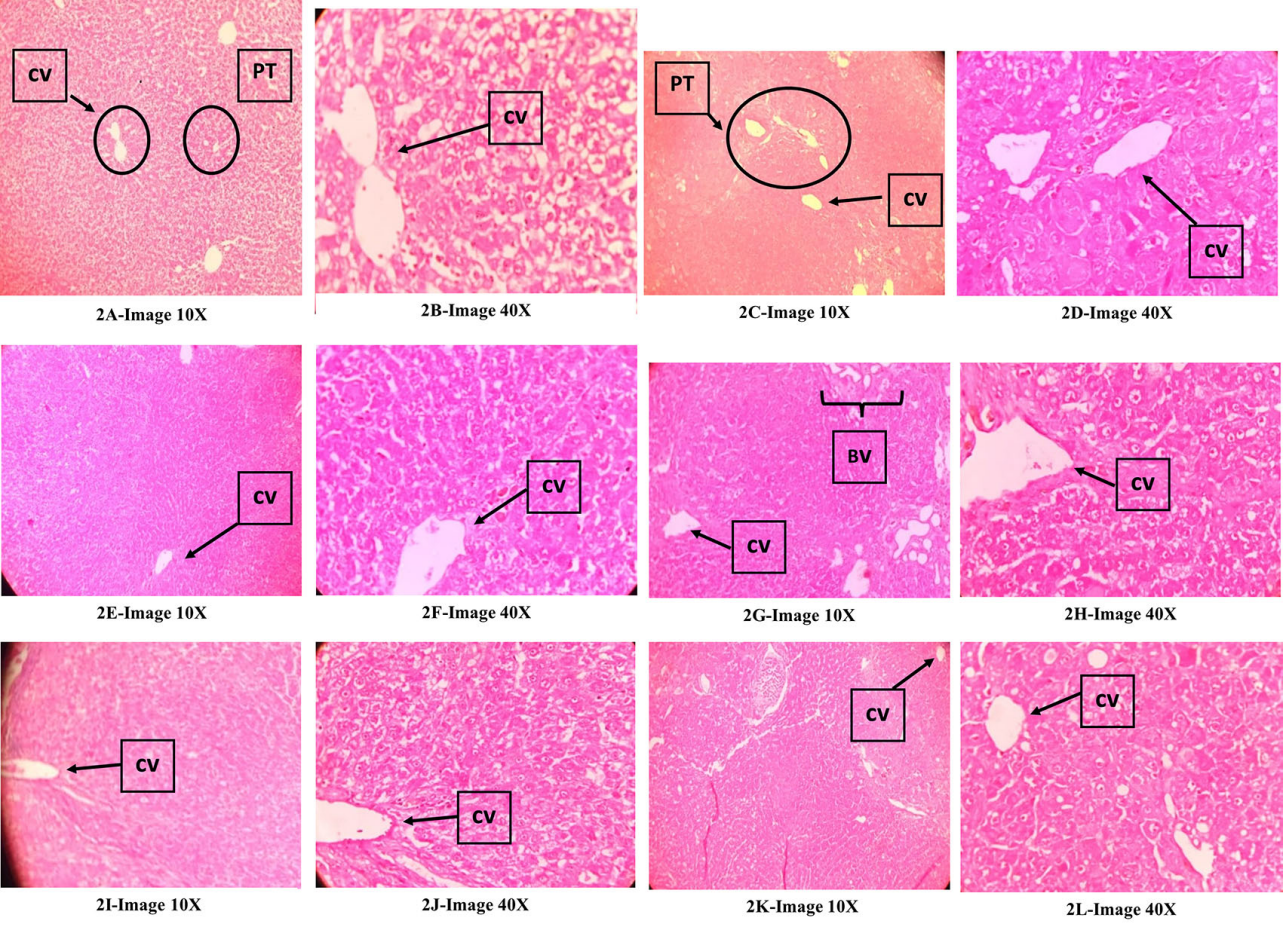
Effect of KM on histopathology of the liver in DEN-induced HCC model in rats; Qualitative assessment of ‘haematoxylin and eosin (H & E) stained liver tissue sections’ were observed under 10× and 40× magnifications; CV-Central vein, BV-Blood vessels, PT-Portal triad.

Figure 2A, 2B (normal control): Showed normal liver structure, Figure 2C, 2D (DEN control) **:** Showed an invasive tumor involving irregular nodules divided by thin fibrous septae and composed of solid sheets and broad trabeculae of neoplastic hepatocytes with round to polygonal cells, irregular nuclei, prominent nucleoli, vesicular chromatin, moderate to abundant granular eosinophilic cytoplasm, mitotic figures, scattered with numerous variably dilated sinusoids, flattened endothelial cells and focal bile pigments. The presence of many areas of high vascularity with several groups of cut sections of blood vessels and destruction of the typical pattern of hepatic cords/hepatocyte arrangement is indicative of hepatocellular carcinoma. Figures 2E, 2F (DEN+KM36), Figures 2G, 2H (DEN+ KM72), Figures 2I, 2J(DEN+KM144 **):** The structure of liver looked normal with hexagonal hepatic lobules, a central vein in its center. However, there were areas showing features of HCC like well-vascularized tumours with thick trabeculae, distinct acinar pattern, some areas of vascular invasion, and signs of mild cytoplasmic vacuolar degeneration. However, the severity of the features of HCC in this group was very mild when compared with that of DEN. Figures 2K, 2L (DEN+ Silymarin): There were areas of mild cytoplasmic vacuolar degeneration and high vascularity indicative of HCC. But it was less when compared with that of DEN group. In general, there were normal liver tissues in large areas compared to the areas with features of HCC.

### Polyacrylamide Gel Electrophoresis for the separation of proteins and mass spectrometry for the identification of proteins

**Figure 3.**
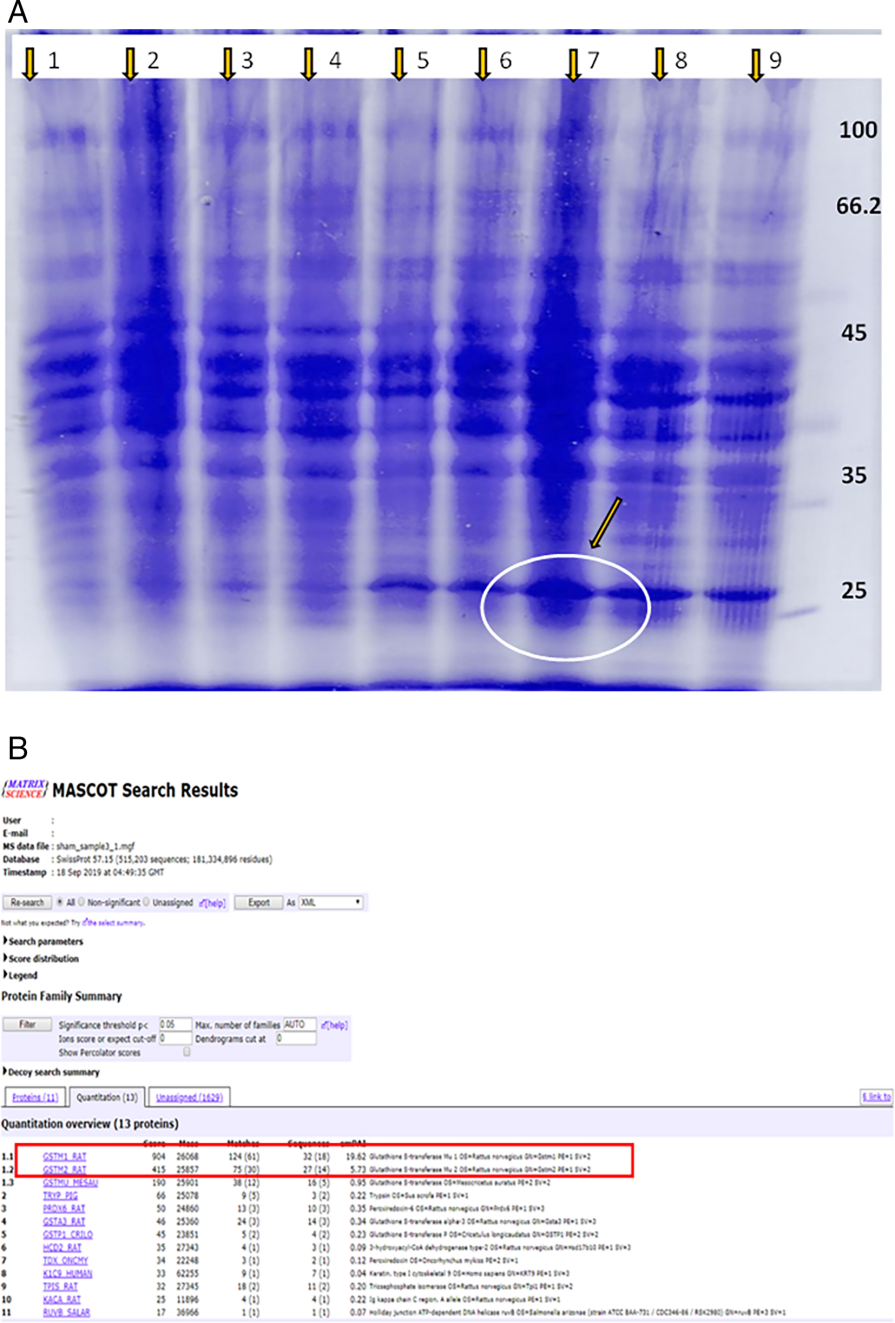
(A) Agarose gel electrophoresis of extraction from the liver of rats; (B) Mass spectrometry analysis of extraction from the liver of rats.

Ladder: Lanes (1–4) - normal control and test drug control groups; Lane 5 - DEN alone, Lanes 6–9 - DEN for 16 weeks followed by treatment with KM 36, 72 and 144 mg/kg, and silymarin, respectively. The protein lysate was prepared and subjected to separation by PAGE ( Figure 3A). DEN control group had a band in the range of 25-30 kda as analysed by Polyacrylamide Gel Electrophoresis (PAGE). In comparison to lanes 5 and 6, band thickness was more in lanes 7, 8, and 9; this was confirmed by mass spectrometry and identified as GSTM1 ( Figure 3B).

### In-silico analysis of interactions of phytoconstituents of KM with β catenin

**Figure 4.**
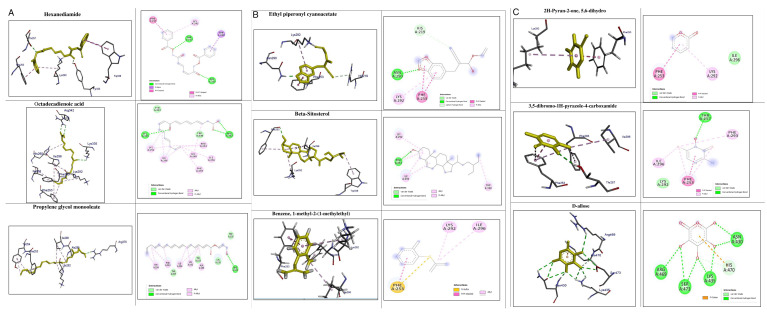
Representation of the interactions made by the shortlisted phytoconstituents of KM with the binding site of β catenin. (Left: binding poses; Right: 2D images) (< 5 Å distance).

The molecular docking study revealed that nine phytoconstituents out of 19 made interactions with β catenin binding site that is residue number 219 to 335 (C-terminal helical region of Tcf-4interaction region) except D-allose which showed interactions between the residues 429 to 508 (extended region of Tcf-4 peptide interaction region) of β catenin. Their binding energies, number of interactions, and interacting residues of β catenin were tabulated in Table 3. The binding energies of the phytoconstituents ranged from -7.8 to -5.2 kcal/mol. The lowest binding energy was recorded for hexanediamide whereas, 2H-Pyran-2-one, 5,6-dihydro documented the highest among the shortlisted constituents. The phytoconstituents with interacting residues are displayed in Figure 4A and 4B as follows. Octadecadienoicacid and propylene glycol monooleate made a maximum number of interactions (8 each) whereas, Hexanediamide made the lowest number of interactions- 2 ( Table 3). However, the constituents did not show any favorable interactions with the interaction site of LRP6-E3.

**Table 3.** Shortlisted phytoconstituents and their interactions with β catenin.

Phytoconstituents	Binding energy	No. of interactions	Hydrogen bonds	Hydrophobic bonds
Hexanediamide	-7.8	2	-	2(Phe253, lys252)
Octadecadienoicacid	-6.0	8	Lys335	7 (His260, Ile296, Lys292, Phe253)
Propylene glycol monooleate	-6.6	8	Arg376	7 (Tyr254, Phe253, Ile296, Lys292)
Ethyl piperonylcyanoacetate	-6.4	4	Asn290	3 (His219, Phe253, Lys292)
Beta-sitosterol	-7.3	5	Phe253	4(Ile296, Lys292, phe253)
Benzene, 1-methyl-2-(1-methylethyl)	-5.7	4	**-**	4 (Ile296, Lys292, Phe253)
2H-Pyran-2-one, 5,6-dihydro	-5.2	2	**-**	2 (Lys292, Phe253)
3,5-dibromo-1H-pyrazole-4-carboxamide	-5.5	4	-	4 (Ile296, Phe253)
D-allose	-6.5	4	4 (Lys435, His470)	**-**

### Gas chromatography-mass spectroscopy (GC-MS) analysis of test drug KM ( Figure 5) ( Table 4)

**Figure 5.**
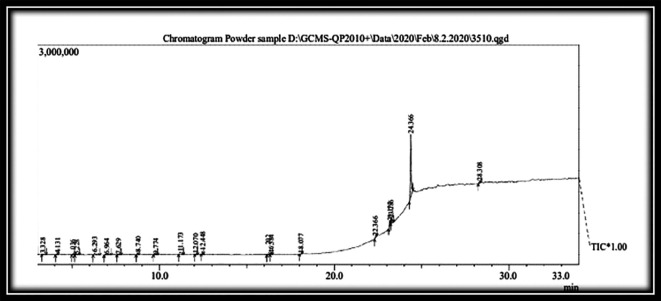
GC-MS analysis of the test drug *Kadukkai maathirai* (KM).

**Table 4.** Gas chromatography-mass spectroscopy (GC-MS) analysis of the test dru *g Kadukkai maathirai* (KM).

Sl. No	R. time	Compound name	Mol. Formula	Mol. Weight	Percentage	Structure
**1**	24.366	Piperine	C _17_H _19_NO _3_	285	66.26	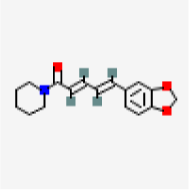
**2**	11.173	D-Allose	C _6_H _12_O _6_	180.16	4.97	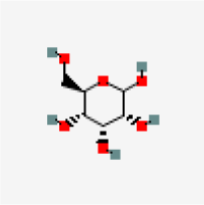
**3**	23.177	Propyleneglycol monooleate	C _21_H _40_O _3_	340	4.68	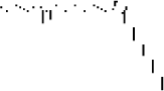
**4**	6.293	Levoglucosenone	C _6_H _6_O _3_	126	3.15	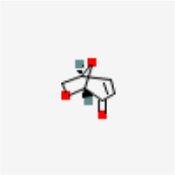
**5**	12.448	Caryophyllenyl alcohol	C _15_H _26_O	222	2.57	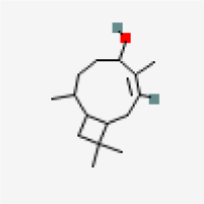
**6**	8.740	Pentadecane	C _15_H _32_	212	2.06	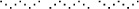
**7**	6.964	Hexanediamide, N,N'-di-benzoyloxy-	C _2_0H _2_0N _2_O _6_	384	2.00	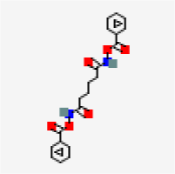
**8**	28.308	beta-Sitosterol	C _29_H _50_O	414	1.72	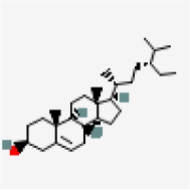
**9**	22.366	Ethyl piperonylcyanoacetate	C _13_H _13_NO _4_	247	1.72	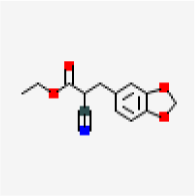
**10**	16.202	10,12-Octadecadienoic acid, 9-oxo-	C _18_H _30_O _3_	294	1.46	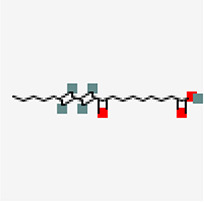
**11**	16.384	n-Hexadecanoic acid	C _16_H _32_O _2_	256	1.37	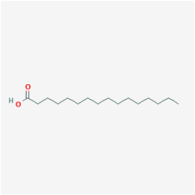
**12**	12.070	Dodecanoic acid	C _12_H _24_O _2_	200	1.18	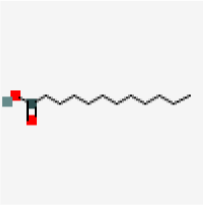
**13**	18.077	cis-9-Hexadecenal	C _16_H _30_O	238	1.13	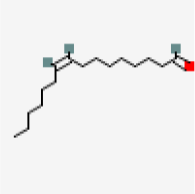
**14**	7.629	1,4:3,6-Dianhydro-.alpha.-d-glucopyranose	C _6_H _8_O _4_	144.1	1.06	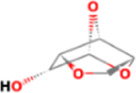
**15**	9.774	3a,7-Methano-3aH-cyclopentacyclooctene, 1,4,5,6,7,8,9,9a-octahydro-1,1,7-trimethyl-, [3	C _15_H _24_	204	1.00	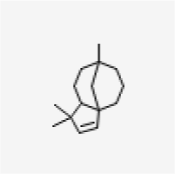
**16**	5.036	Benzene, 1-methyl-2-(1-methylethyl)-	C _1_0H _14_	134	0.94	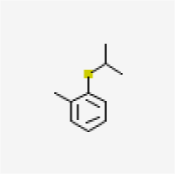
**17**	4.131	3-Aminopyrazine 1-oxide	C _4_H _5_N _3_O	111	0.94	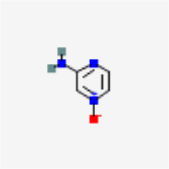
**18**	5.228	2H-Pyran-2-one, 5,6-dihydro-	C _5_H _6_O _2_	98	0.90	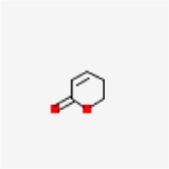
**19**	3.328	Oxime-, methoxy-phenyl-	C _8_H _9_NO _2_	151	0.90	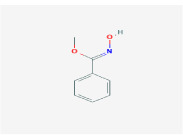

19 compounds were recognized by GC-MS from methanolic extract of *KM.* Piperine (66.26%) was the major constituent of KM. Others were D-Allose (4.97%), Propyleneglycol monoleate (4.68%), Levoglucosenone (3.15%), etc.

## Discussion

N-nitroso compounds, as potential carcinogens, were first brought to the public attention as a cause of liver disease in 1937. In 1954, it was confirmed that one oral or parenteral dose of DEN (20-40 mg/kg) acts as a liver toxin, leading to severe liver necrosis in rodents. ^34^^,^^35^ In rodents, chronic parenteral and oral administration of DEN at large doses effectively induces liver tumour. ^36^ Pericentral foci of small dysplastic liver cells is induced by diethylnitrosamine (DEN), by ethylating DNA ^37^^,^^38^ leading to cirrhosis and multifocal HCC. ^39^ It predominantly targets the liver, where it is biologically transformed by CYP450-dependent processes that are most active in centrilobular hepatocytes. ^40^ The main metabolising pathways of DEN in rodents and humans are very similar. ^41^ which results in reactive species responsible for the development of methylated compounds, leading to carcinogenesis. Previous studies have shown that in DEN-treated group, there was distorted liver architecture with swollen cells, hyperplasia, and dysplasia, with infiltration of inflammatory cells, indicating carcinogenesis. ^42^^–^^44^In the present study, similar histopathological changes occurred in DEN-treated groups.

DEN-induced hepatic injury leads to instability of metabolism by the liver with concomitant variations in the activities of enzymes. ^45^ An increase in the level of transaminases indicates liver damage. Liver toxicity leads to the breakdown of the cell membrane and further spillage of the aminotransferases from the cytoplasm into the serum, thus increasing the serum aminotransferase levels. ^46^Aminotransferases are known as diagnostic and reliable markers of liver damage. In our study, the level of liver enzymes and bilirubin were increased significantly reflecting liver damage in DEN alone treated rats. In rats which received DEN with KM, the enzyme levels were reduced when compared to DEN alone treated rats, though it was not statistically significant. Probably, increasing the duration of treatment (> 4 weeks) with KM may have produced further improvement in liver functions.

Weight loss is an important symptom of DEN-induced cancer. DEN causes alterations which is reflected as worsening of liver function during pathogenesis of liver fibrosis. ^47^ Damaged to the liver results in loss of appetite. Administration of KM in varying doses following induction of HCC by DEN prevented body weight loss which could be due to its protective action against liver damage.

In this study, the relative liver weights of DEN-treated rats were significantly high compared to normal which has been commonly observed and reported in liver cancer. ^48^ Treatment with KM prevented an increase in liver weight. DEN gets metabolized by microsomal enzymes in a sequence of intermediate responses leading to development of mutagenic metabolites. The free radicals generated in the process trigger cell proliferation, appearance of liver nodules and a rise in the weight of the liver. ^49^ Low levels of ROS do not cause any damage to the cell process, but high ROS level damages proteins, lipids and DNA by nonspecific process. ^50^ Glutathione-S transferase mu 1 (GSTM1) is a phase II detoxifying enzyme. It plays a role in the detoxification of environmental carcinogen metabolites, suggesting that its downregulation may contribute to HCC carcinogenesis secondary to ROS-facilitated oxidative damage. ^51^ The expression of GSTM1 was much higher in the KM and silymarin treatment groups versus control and DEN groups.

*Eclipta alba*, a constitiuent of KM has been to shown to have antiproliferative property in HepG2 cell lines. *Eclipta alba* extract could induce DNA fragmentation and thus apoptosis in hepatic cell lines. ^52^Alkaloids present in *Eclipta alba* can damage the DNA of cancer cells. ^53^ *Eclipta alba* is a well-known liver regenerative herb with protective effect on liver parenchymal cells and its cell membrane resulting in a decrease in enzyme seepage. ^54^ An important constituent of *Terminalia chebula* is chebulic acid. ^55^ Chebulic acid has been established to have hepatoprotective and antioxidant effects in various liver disease models. ^56^ In a previous study, *Terminalia chebula* stimulated apoptosis with membrane bleb and apoptotic bodies, thus rendering protection to macromolecules in DEN model by stabilizing the redox balance. ^57^

Piperine, present in *Piper nigrum of Kadukkai maathirai* has demonstrated dose-dependent cytotoxicity against HepG2 cells. It inhibits catalase and induces mitochondria-mediated cell death by H _2_O _2_ in these cells. Further, analysis revealed that it had receptor tyrosine kinase inhibition property which led to inhibition of HCC progression. ^58^ Glucose, a major source of energy for cancer cells, is transported into cells via glucose transporters (GLUTs). These transporters are overexpressed in cancer cells which helps in enhanced glucose uptake by the cancer cells. Studies have shown that D-allose inhibited GLUT1 expression in cell lines of hepatocellular carcinoma thus reducing glucose utilization by cancer cells. ^59^

Citrus fruits contain valuable bioflavonoids that have bioactivities on apoptosis induction *in vitro.* ^60^ The phytochemicals present in citrus fruits exhibit antioxidant, anticancer, and anti-inflammatory activities. ^61^^–^^63^ The constituent plants of KM could have exerted synergistic effects to protect against DEN induced hepatic damage.

Constitutive Wnt signalling has been document as a key factor in the development of various cancers. β catenin is a multifunctional protein in Wnt signalling pathway. Transcription factor 4 (Tcf4) is a peptide that interacts with the armadillo repeat region of β catenin leading to production of downstream transcription factors for malignant growth. The Tcf4 interaction regions on β catenin have been studied and used as templates for analysis. ^64^^,^^65^ Among the phytoconstituents of KM, D-allose contributed a maximum number of hydrogen interactions-4 with Lys435, His470. Octadecadienoicacid and propylene glycol monooleate showed the maximum number of interactions of eight with one hydrogen interaction. All other phytoconstituents showed interactions with this region of β catenin. With this analysis, we hypothesize that these constituents could interact with this region simultaneously or in part, thus potentially modulating the transcription process by preventing Tcf4 interaction with β catenin. We also noted that the shortlisted constituents would not modulate the LRP6-DKK1 mediated Wnt stimulation and subsequent pathway activation as they did not make any interactions with the LRP6-E3 binding site. In the present study beta sitosterol was confirmed in the GCMS analysis of KM. Earlier studies have reported that beta sitosterol downregulates beta catenin expression inhibits growth and induces apoptosis in colon cancer cells. ^66^ We presume that the active phytoconstituents of *Kadukkai maathirai* could modulate the intracellular transcription process by inhibiting the armadillo repeat region of β catenin.

## Conclusion

We have concluded that *Kadukkai maathirai* could be potentially useful as a supplement in therapy of hepatocellular carcinoma. Further studies are needed to investigate its mechanism of action including validation of its target in hepatocellular carcinoma before exploring its efficacy in clinical studies.

## Ethics statement

Seven-week-old female Sprague Dawley rats (150-200 g) procured from the Central Animal Research Facilities (CARF) of Kasturba Medical College, Manipal, Manipal Academy of Higher Education, Manipal were used in the study after getting approval from the Institutional Animal Ethics Committee approval (IAEC/KMC/19/2016 dated 16.03.2016). Guidelines given by the Committee for Control and Supervision of Experiments on Animals, Government of India, New Delhi for the use of laboratory animals were followed for the maintenance of animals. ^18^

## Data Availability

• Figshare: Macroscopic appearance of the liver https://doi.org/10.6084/m9.figshare.25117424.v1 ^67^
• Figshare: Histopathology https://doi.org/10.6084/m9.figshare.25117436.v1 ^68^
• Figshare: Polyacrylamide Gel Electrophoresis for the separation of proteins and mass spectrometry for the identification of proteins https://doi.org/10.6084/m9.figshare.25117442.v1 ^69^
• Figshare: In-silico analysis of interactions of phytoconstituents of KM with β catenin https://doi.org/10.6084/m9.figshare.25117454.v1 ^70^
• Figshare: Gas chromatography-mass spectroscopy (GC-MS) analysis of test drug KM https://doi.org/10.6084/m9.figshare.25117460.v1 ^71^
• Figshare: Effect of KM on liver function in DEN-induced HCC model in rats https://doi.org/10.6084/m9.figshare.25117469.v1 ^72^
• Figshare: Effect of “KM on body weight, relative weight of liver and nodule count in DEN-induced hepatocellular carcinoma model in rats https://doi.org/10.6084/m9.figshare.25117484.v1 ^73^
• Figshare: Shortlisted phytoconstituents and their interactions with β catenin https://doi.org/10.6084/m9.figshare.25117490.v1 ^74^
• Figshare: Gas chromatography-mass spectroscopy (GC-MS) analysis of the test drug Kadukkai maathirai (KM) https://doi.org/10.6084/m9.figshare.25117493.v1 ^75^
• Figshare: Raw Data https://doi.org/10.6084/m9.figshare.25815934.v1 ^76^ Figshare: ARRIVE checklist for *Kadukkai maathirai* (Indian herbal drug) prevents hepatocellular cancer progression by enhancing GSTM1 expression and modulating β catenin transcription: in-silico and in-vivo study https://doi.org/10.6084/m9.figshare.25910563.v1 ^77^ (Shetty MS. ARRIVE CHECKLIST [Internet]. figshare; 2024 [cited 2024 Jun 7]. Available from: https://figshare.com/articles/dataset/ARRIVE_CHECKLIST/25910563/1) Data are available under the terms of the Creative Commons Attribution 4.0 International license (CC-BY 4.0).
