# Supplementary material for: Kadukkai maathirai (Indian herbal drug) prevents hepatocellular cancer progression by enhancing GSTM1 expression and modulating β catenin transcription: in-silico and in-vivo study
Source: F1000Res. 2024 Oct 22;13:639. Originally published 2024 Jun 17. [Version 2] doi: 10.12688/f1000research.145961.2 (PMC11800331; doi:10.12688/f1000research.145961.2)
Supplement: Peer review history

# Reviewer report: Reviewer response for version 2

**DOI:** 10.5256/f1000research.172786.r345193

**Published:** 2025-01-09

**Authors:** Afrah Fatthi Salama

**Affiliations:** 1 Professor of Biochemistry, Faculty of Science, Tanta University, Tanta, Gharbia Governorate, Egypt

- Page 5, under Animals, please add Reference for using female rats, giving reason for using female not male 

- You gave DEN, in drinking water as Reference no. 20, this reference used male rats? Is that effect applied on female? And do you calculate the dose that every female rat received for tumor induction? 

- Page 7, Table 2. Relative liver weight, how calculated?, you put (g)?, it have to be (g/100 g Body weight), okay. Also Body weight (g), what does it mean? Is that the final body weight?. It have to be (Body weight gain, which is the difference between animal weight at the beginning and at the end of the experiment , to be positive or negative number), please correct it as I explained. 

- The same table 2. Nodule count, how can you do this count?. I noticed that the dose 36 mg/kg of KM is the best, than larger ones, also silymarin give the lowest number of nodule count  than all doses of KM, and you have to explain these results well in the paper 

- In fig. 1. why you did not mark the position of nodules like the other groups? 

- Write Glutathione S- Transferase , like this  

- Finally Results , Discussion and conclusion need more explanation for all results. 
  Best Regards

Is the work clearly and accurately presented and does it cite the current literature?

Partly

If applicable, is the statistical analysis and its interpretation appropriate?

I cannot comment. A qualified statistician is required.

Are all the source data underlying the results available to ensure full reproducibility?

Yes

Is the study design appropriate and is the work technically sound?

Yes

Are the conclusions drawn adequately supported by the results?

Partly

Are sufficient details of methods and analysis provided to allow replication by others?

Yes

Reviewer Expertise:

Clinical Biochemistry- Natural products –Cancer Biology-Toxicology, and my Search inter in applied fields, see it on google scholar

I confirm that I have read this submission and believe that I have an appropriate level of expertise to confirm that it is of an acceptable scientific standard, however I have significant reservations, as outlined above.

# Reviewer report: Reviewer response for version 1

**DOI:** 10.5256/f1000research.159988.r309884

**Published:** 2024-08-21

**Authors:** Talha Bin Emran

**Affiliations:** 1 BGC Trust University Bangladesh, Chittagong, Chittagong Division, Bangladesh

1. The results and discussion section could benefit from a more detailed exploration of the findings. It would be helpful to provide additional insight into why certain effects are coming in to existence and what could be the possible reason behind them? 

 2. Results and Conclusion: The section devoted to the explanation of the results suffers from the same problems revealed so far. The storyline in the results section (and conclusion) is hard to follow. Moreover, the conclusions presented appear to diverge from the insights suggested by the empirical results. It may be beneficial to revisit and align the conclusions more closely with the findings.

 3. The discussion should be rather organized around arguments avoiding simply describing details without providing much meaning. A real discussion should also link the findings of the study to theory and/or literature.

Is the work clearly and accurately presented and does it cite the current literature?

Partly

If applicable, is the statistical analysis and its interpretation appropriate?

Partly

Are all the source data underlying the results available to ensure full reproducibility?

Partly

Is the study design appropriate and is the work technically sound?

Partly

Are the conclusions drawn adequately supported by the results?

Yes

Are sufficient details of methods and analysis provided to allow replication by others?

Yes

Reviewer Expertise:

Postdoctoral scientist with 2+ years of experience in viral and non-viral gene editing, particularly using the CRISPR/Cas9 system and DNA Damage Repair (DDR) in cancer biology. Proficient in handling mammalian cancer cells, conducting cell viability assays, and performing advanced molecular techniques such as next-generation sequencing. Actively engaged in researching DDR gene targets to overcome cancer therapy resistance. Prepared to contribute to the academic, pharmaceutical, biotech, and translational research sectors, focusing on gene therapy, gene editing, immune-oncology, mRNA, oncological biomarkers, and the development of small molecule/DDR-targeted inhibitors. Possesses a strong foundation in the practical applications and potential of small molecule inhibitor screening.

I confirm that I have read this submission and believe that I have an appropriate level of expertise to confirm that it is of an acceptable scientific standard, however I have significant reservations, as outlined above.

# Reviewer report: Reviewer response for version 1

**DOI:** 10.5256/f1000research.159988.r309885

**Published:** 2024-08-21

**Authors:** Haiyang Yu

**Affiliations:** 1 Tianjin University of Traditional Chinese Medicine, Tianjin, China

1. This article demonstrates that KM can protect the liver from further damage by reducing the nodule count and reducing the relative weight of the liver. Also, this paper verifies that KM regulates the intracellular transcription process by reducing GSTM1 expression and simultaneously inhibiting the armadillo repeat region of β catenin to realize the potential of inhibiting HCC. But the hepatoprotection mechanism of KM against diethylnitrosamine-induced liver cancer needs to be further improved. It suggest that the author should further study and explain.
2. The arrangement of the pictures, tables, and text in the article is too messy. For example, Figure 5 and Table 4, it is recommended to arrange them separately. We recommended that the picture and tables should be placed above or below the page for reading.
3. In some similar paper, some cell experiments will be done to verify the results obtained in animal experiments. We suggest that the author can do some cell experiments to further study.
4. Page 16, conclusions, “Hence, it could be concluded that the collective effect of all the herbs present in KM was responsible for its beneficial effect.” The description is just too absolute.
5. Please unify the cell line names included in the article. For example, page 15，HepG2 cell and Hep G2 cells.

Is the work clearly and accurately presented and does it cite the current literature?

Yes

If applicable, is the statistical analysis and its interpretation appropriate?

Yes

Are all the source data underlying the results available to ensure full reproducibility?

Yes

Is the study design appropriate and is the work technically sound?

Yes

Are the conclusions drawn adequately supported by the results?

Yes

Are sufficient details of methods and analysis provided to allow replication by others?

Yes

Reviewer Expertise:

hepatocellular cancer progression

I confirm that I have read this submission and believe that I have an appropriate level of expertise to confirm that it is of an acceptable scientific standard.
